# High‐Speed Centrifugation Efficiently Removes Immunogenic Elements in Osteochondral Allografts

**DOI:** 10.1111/os.13991

**Published:** 2024-01-18

**Authors:** Yongsheng Ma, Qitai Lin, Wenming Yang, Yang Liu, Yugang Xing, Zhiyuan Ren, Xueding Wang, Raorao Zhou, Gaige Wu, Pengcui Li, Wangping Duan, Xiaoling Zhang, Xiaochun Wei

**Affiliations:** ^1^ Department of Orthopaedics Second Hospital of Shanxi Medical University Taiyuan China; ^2^ Shanxi Key Laboratory of Bone and Soft Tissue Injury Repair Taiyuan China; ^3^ Department of Orthopedic Surgery Xin Hua Hospital Affiliated to Shanghai Jiao Tong University School of Medicine Shanghai China

**Keywords:** Allografts, Articular Cartilage, Centrifugation, Immunogenic Elements, Pulse Lavage

## Abstract

**Objectives:**

The current clinical pulse lavage technique for flushing fresh osteochondral allografts (OCAs) to remove immunogenic elements from the subchondral bone is ineffective. This study aimed to identify the optimal method for removing immunogenic elements from OCAs.

**Methods:**

We examined five methods for the physical removal of immunogenic elements from OCAs from the femoral condyle of porcine knees. We distributed the OCAs randomly into the following seven groups: (1) control, (2) saline, (3) ultrasound, (4) vortex vibration (VV), (5) low‐pulse lavage (LPL), (6) high‐pulse lavage (HPL), and (7) high‐speed centrifugation (HSC). OCAs were evaluated using weight measurement, micro‐computed tomography (micro‐CT), macroscopic and histological evaluation, DNA quantification, and chondrocyte activity testing. Additionally, the subchondral bone was zoned to assess the bone marrow and nucleated cell contents. One‐way ANOVA and paired two‐tailed Student's t‐test are used for statistical analysis.

**Results:**

Histological evaluation and DNA quantification showed no significant reduction in marrow elements compared to the control group after the OCAs were treated with saline, ultrasound, or VV treatments; however, there was a significant reduction in marrow elements after LPL, HPL, and HSC treatments. Furthermore, HSC more effectively reduced the marrow elements of OCAs in the middle and deep zones compared with LPL (*p* < 0.0001) and HPL (*p* < 0.0001). Macroscopic evaluation revealed a significant reduction in blood, lipid, and marrow elements in the subchondral bone after HSC. Micro‐CT, histological analyses, and chondrocyte viability results showed that HSC did not damage the subchondral bone and cartilage; however, LPL and HPL may damage the subchondral bone.

**Conclusion:**

HSC may play an important role in decreasing immunogenicity and therefore potentially increasing the success of OCA transplantation.

## Graphical abstract

The subchondral bone in osteochondral allografts contains many bone marrow cells, blood cells, lipids, and proteins, which are mostly immunogenic. High‐speed centrifugation (HSC) efficiently removes immunogenic elements. HSC could play an important role in decreasing immunogenicity and therefore has the potential to increase success.

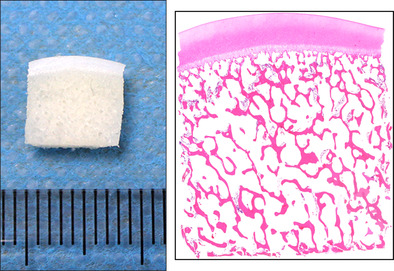

## Introduction

Articular cartilage (AC) defects are a common disorder affecting people of all ages; however, their treatment remains challenging.^1^ As cartilage lacks nerves, lymphatic vessels, and blood vessels, it is unable to heal itself.^2^, ^3^ In the aging population, cartilage or osteochondral lesions can be successfully treated by arthroplasty; however, arthroplasty is not acceptable for young and active patients.^4^, ^5^ Alternatively, fresh osteochondral allograft (OCA) transplantation has an extensive clinical history of repairing osteochondral defects, with 10‐year survival rates of over 75%.^5^, ^6^, ^7^, ^8^ The success of fresh OCA transplantation relies on the provision of viable cartilage and stable subchondral bone; however, failure can still occur. The main point of failure in OCA transplantation failure is the bone component, often manifesting as graft collapse, subchondral bone cyst, non‐healing of the graft interface, and abnormal pain.^9^, ^10^, ^11^, ^12^, ^13^ Several studies have reported that graft‐host immune rejection may be a potential cause of OCA transplantation failure.^5^, ^12^, ^14^, ^15^, ^16^, ^17^

The main factors contributing to the immunogenicity of grafts are allograft cartilage and subchondral bone.^18^ Chondrocytes have immunogenic elements; however, the special composition and structure of the extracellular matrix protect them from the recognition of chondrocyte surface antigens by the host immune system, thereby allowing them to successfully avoid rejection.^4^, ^15^ Additionally, the subchondral bone contains many bone marrow cells, blood cells, lipids, and proteins, which are mostly immunogenic.^18^, ^19^ Currently, pulse lavage (PL) is used clinically to remove bone marrow elements from the subchondral bone of OCAs as much as possible to reduce immune rejection occurrence.^20^, ^21^, ^22^, ^23^, ^24^ However, the effectiveness of PL has been questioned; it did not significantly reduce the bone marrow content of OCAs, possibly because it could not remove the deep bone marrow elements.^19^ Thus, the removal of immunogenic elements of OCAs requires better methods during pre‐transplantation.

In this study, we hypothesized that five such methods could physically remove the immunogenic elements of OCAs. The vortex vibration (VV) method removes blood and lipids from the graft by inducing an oscillatory effect through high‐speed rotation; the ultrasound method uses high‐frequency vibrations to remove immunogenic elements from bone tissue and pore surfaces;^25^ and the centrifugation method uses the centrifugal force of high‐speed rotation to separate the blood, bone marrow, and lipids from the inside of the graft.^26^, ^27^

Hence, the purpose of the study was to (i) validate whether the various methods were feasible, whether the chondrocyte viability and extracellular matrix were affected, and whether trabecular bone structure was destroyed after treatment of OCAs and to (ii) evaluate the effectiveness of each method for removing immunogenic elements from OCAs to identify an optimal method.

## Materials and Methods

### Graft Harvest and Preservation

Porcine knees weighing approximately 150 kg from skeletally mature pigs were collected from a local abattoir immediately after slaughter; they were immediately transported to the laboratory following examination. Twenty‐four knee joints were obtained. OCAs were obtained from the femoral condyle of each joint using aseptic techniques. If there was obvious arthritis such as profound fibrillation or cartilage loss, the joints were omitted. OCAs were excluded if the cartilage was separated from the subchondral bone or the subchondral bone was broken. Six grafts were obtained from each joint using an OCA transplant instrument (Smith & Nephew Inc. USA), totaling 143 OCAs (8.5 mm diameter, subchondral bone of 6 mm thickness) that could be used in the experiments.^28^, ^29^, ^30^

OCAs were collected and preserved for 3 days in Dulbecco's Modified Eagle Medium/F12 (Gibco, USA) with penicillin and streptomycin (100X) (Meilunbio, China). The OCAs were processed under sterile conditions. Subsequently, OCAs were distributed randomly into seven groups: (1) control group (n = 17), (2) saline group (*n* = 21), (3) ultrasound group (*n* = 21), (4) VV group (*n* = 21), (5) low‐pulse lavage (LPL) group (*n* = 21), (6) high‐pulse lavage (HPL) group (*n* = 21), and (7) high‐speed centrifugation (HSC) group (*n*= 21) (Table 1).

**TABLE 1:** Study groups and treatment methods

Group	Treatment
Control (*n* = 17)	Fresh OCAs, no treatment
Saline (*n* = 21)	The OCA was rinsed with sterile saline for 2 min and rotated every 10 s. Per plug, 1 L saline was used
Ultrasound (*n* = 21)	The OCA was placed in 50‐mL centrifuge tubes with 30 mL of sterile saline and underwent high‐frequency ultrasound treatment (Scientz, Ningbo, China): frequency of 40 kHz and 2 min time.
VV (*n* = 21)	The OCA was placed in 50‐mL centrifuge tubes with 30 mL of sterile saline and shaken for 2 min (3000 r/min) using a vortex mixer (Thermo Scientific, Massachusetts, USA).
LPL (*n* = 21)	The OCA was irrigated using conventional pulse lavage equipment (Clean, Guangzhou, China). The sample was rinsed with saline as close to the spray tip as possible, from the base of the subchondral bone upwards, rotating every 10 s, for 2 min, at a flow rate of 1100 mL/min.
HPL (*n* = 21)	Same as the method described above, with a lavage flow rate of 1260 mL/min.
HSC (*n* = 21)	The OCA was placed into 1.5‐mL centrifuge tubes and centrifuged for 2 min at 12000 r/min (5430R, Eppendorf, Germany).

Abbreviations: OCAs, osteochondral allografts; VV, vortex vibration; LPL, low‐pulse lavage; HPL, high‐pulse lavage; HSC, high‐speed centrifugation.

### Weight Assessment

For each control and experimental group, five OCAs were assessed for dry weight as an indicator of the weight of bone marrow elements after removal. The OCAs were collected and immediately frozen at −80°C for 4 hours, placed in a vacuum freeze drier (Alpha 1–4, Christ, Germany), and then processed according to standard drying procedures. After 24 h, the OCAs were measured using an electronic balance.

### Micro‐Computed Tomography (micro‐CT) Evaluation

For the six experimental groups, both pre‐and post‐treatment samples were scanned by a micro‐CT system (vivaCT80, SCANCO Medical AG, Switzerland) at high resolution with four OCAs per group.^31^ OCAs were scanned, and successive micro‐CT two‐dimensional (2D) images were obtained with an image resolution of 1024 × 1024 with a pixel size of 20 × 20 μm, and a layer spacing of 15.6 μm. The subchondral bone was defined as a region of interest, and the images were analyzed using Scanco software. The bone volume per tissue volume (BV/TV, %), connection density of the bone trabeculae (Conn.D, 1/mm^3^), trabecular number (Tb. N, 1/mm), trabecular space (Tb. Sp, mm), and bone mineral density (BMD) (mg HA/ccm) were analyzed.^32^

### Macroscopic Evaluation

For each control and experimental group, the gross appearance of four OCAs was observed to evaluate cleansing efficacy. The OCAs were fixed in 4% paraformaldehyde for 48 h and decalcified using 10% EDTA; after 1 week, the OCAs were evenly divided into two groups using a scalpel and evaluated for gross appearance.

### Histological Analyses

**FIGURE 1:**
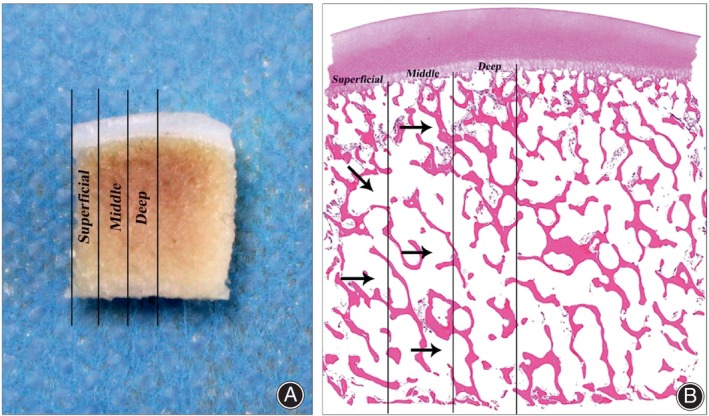
Vertical section of the OCA. (A) Superficial (external third), middle (middle third), and deep (central third) zones identified on the fresh OCA. (B) Pathological sections of the OCA after HSC treatment. The black arrows point to the bone marrow cavity and the trabecular bone and bone marrow cells are shown in red. HSC, high‐speed centrifugation; OCA, osteochondral allograft.

For each control and experimental group, four OCAs were histologically analyzed to evaluate the effectiveness of the removed bone marrow elements and the completeness of the cartilage. As described above, the OCAs were evaluated macroscopically and subjected to EDTA decalcification. One month later, the OCAs were dehydrated, embedded in paraffin, and sectioned at 5‐mm intervals according to recognized histopathological standards. Sections were stained with hematoxylin and eosin (HE) (Solarbio, Peking, China) to analyze the effectiveness of the removed bone marrow elements in the subchondral bone, with safranin O and fast green (Saf O) (Solarbio, Peking, China) used to assess glycosaminoglycan (GAG) in the cartilage, and Sirius red (Solarbio, Peking, China) used to detect collagen (COL) in the cartilage. Each sample was divided into the following three zones for analysis: superficial (external third), middle (middle third), and deep (central third) (Figure 1A).^19^ The effectiveness of removed bone marrow elements was expressed as a “blank cavity area ratio” technique (Figure 1B). The blank cavity area ratio, which is the area of the blank bone marrow cavity within the bone trabeculae as a percentage of the total area of the bone trabeculae and bone marrow cavity, was calculated using ImageJ software (version 1.51 k).^23^

### DNA Quantification

For each control and experimental group, four subchondral bones were examined for DNA concentration to determine the number of remaining nucleated cells.^19^ The tissue was obtained from the superficial, middle, and deep zones of the subchondral bone, each sample weighing 30 mg. After cutting and grinding, all tissues were incubated in 20 μL lysis buffer and shaken gently at 56°C for 3 hours. Subsequently, DNA was extracted according to the manufacturer's instructions of the TIANamp Genomic DNA Kit (Tiangen, Peking, China) as follows: 200 μL of buffer solution was added, mixed thoroughly, and incubated at 70°C for 10 min to allow the solution to become clear. Later, 200 μL of anhydrous ethanol was added and mixed again. The resulting solution and precipitate were moved into the adsorption column, and the specific solution was repeatedly added to rinse. Finally, 100 μL buffer was added to obtain DNA. The DNA concentration was measured using a NanoDrop One spectrophotometer (Thermo Scientific, Massachusetts, USA) at a wavelength of 260 nm. Nucleic acid purity was assessed using the absorbance ratio A260/A280 nm.^19^

### Chondrocyte Viability

For each control and experimental group, four OCAs were stained for live and dead cells to assess chondrocyte viability.^33^, ^34^ The subchondral bone was removed from the graft using a scalpel. The cartilage was adhered to the specimen table and sectioned in cross‐section using a vibratome (VT 1200S, Leica, Germany) to obtain 50‐um thickness cartilage slices. Each cartilage slice was stained for two‐color fluorescence; the live cell stain calcein‐AM (green color, Bestbio, China) and the dead stain propidium iodide (PI, red color, Bestbio, China) were used for 40 minutes in a water bath at 37°C. Cartilage slices in the deep zone were imaged using a confocal laser‐scanning microscope (Leica, Germany). The ImageJ software was then used to count the cells in the images, and chondrocyte viability was calculated as the percentage of live cells relative to the total number of cells. In each cartilage slice, the chondrocyte viability was determined as the percentage of green cells relative to the total number of green and red cells.^28^, ^35^ The experimental procedures were repeated two times.

### Statistical Analyses

The data are presented as the mean ± standard deviation and statistically analyzed using SPSS 25.0 (IBM, NY, USA) and GraphPad Prism 9 (Prism, CA, USA). Weight assessment, histological analyses, DNA quantification, and chondrocyte viability of OCAs were compared between the control and experimental groups using a one‐way ANOVA of variance with a Bonferroni's *post hoc* test. Micro‐CT evaluations of both pre‐and post‐treatment of OCAs were compared using a paired two‐tailed Student's T‐test. Statistical significance was set at *p* < 0.05.

## Results

### Weight Assessment

**FIGURE 2:**
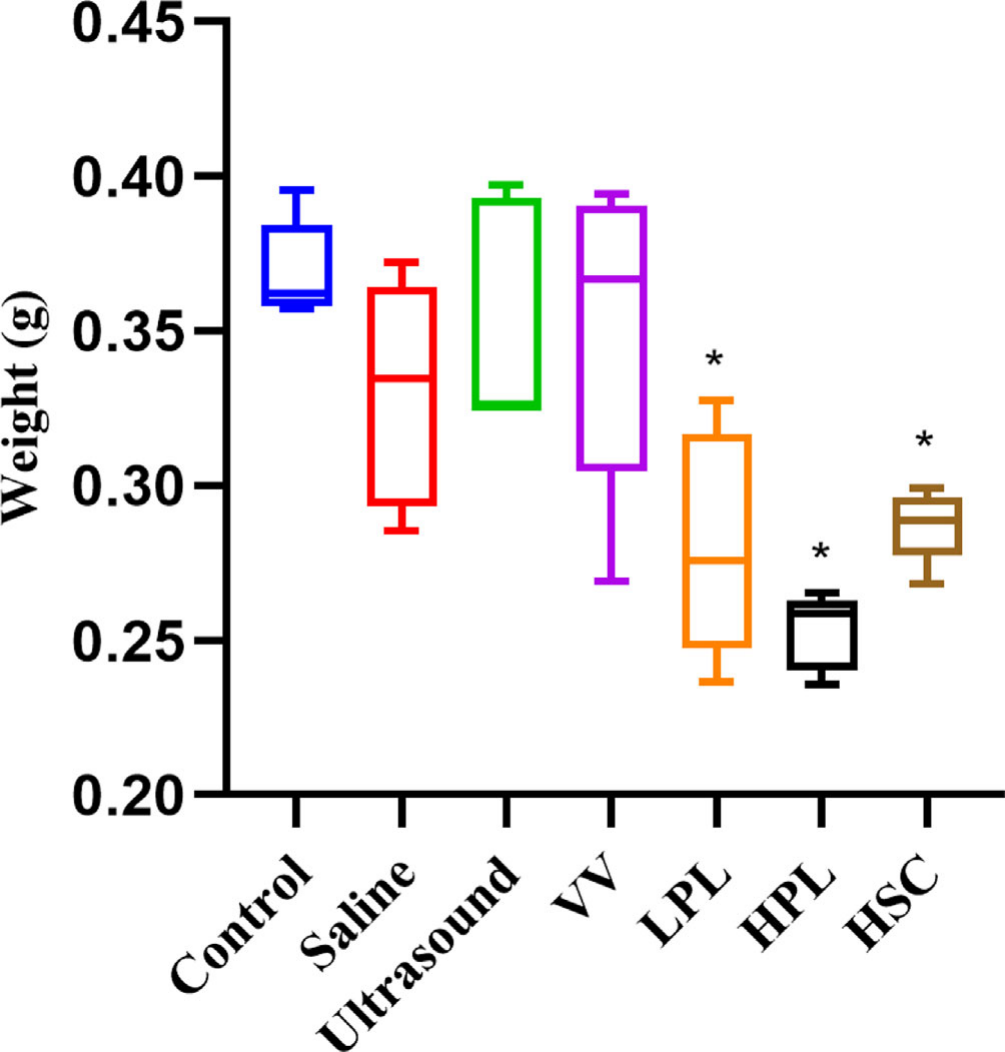
Weight variation of OCAs. Data are reported in box plots with mean, quartiles, and minimum and maximum values. Statistical analysis was performed using one‐way analysis of variance comparing the control and each treatment group with Bonferroni's post‐hoc test. *p < 0.05. HPL, high‐pulse lavage; HSC, high‐speed centrifugation; LPL, low‐pulse lavage; OCAs, osteochondral allografts; VV, vortex vibration.

In the dry weight assessment, there were no significant differences in the saline, ultrasound, and VV‐treated OCA groups compared to the control group; however, significant differences were observed in the LPL (*p* = 0.0009), HPL (*p* < 0.0001), and HSC (*p* = 0.0021) groups. The HPL (0.25 ± 0.12 g) group had the lowest dry weight of OCAs, followed by the LPL (0.28 ± 0.04 g) and HSC (0.29 ± 0.01 g) groups (Figure 2).

### Micro‐CT Findings

**FIGURE 3:**
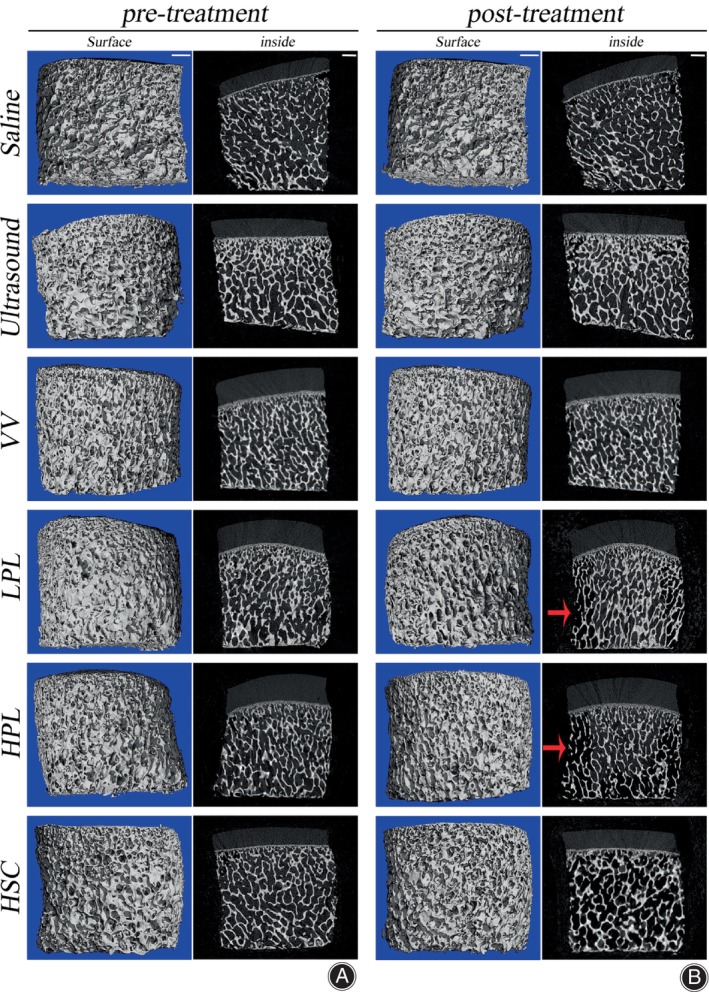
Micro‐Computed Tomography Evaluation of OCAs. (A) The 3D and 2D pictures of pre‐treatment OCAs. Left‐side scale bar = 1000 μm and right‐side scale bar = 1000 μm. (B) The 3D and 2D pictures of post‐treatment OCAs. The red arrows point to sparse bone trabeculae. Left‐side scale bar = 1000 μm and right‐side scale bar = 1000 μm. HPL, high‐pulse lavage; HSC, high‐speed centrifugation; LPL, low‐pulse lavage; OCAs, osteochondral allografts; VV, vortex vibration.

The micro‐CT results from the six experimental groups were analyzed (Table 2). We observed no significant differences in the pre‐and post‐treatment CT results in the saline, ultrasound, VV, or HSC groups; however, significant differences were observed in the pre‐and post‐treatment CT results in the LPL and HPL groups. Additionally, there were significant differences in the pre‐and post‐treatment results between BV/TV (*p* = 0.0494), Tb. N (*p* = 0.0060), and Tb. Sp (*p* = 0.0145) in the LPL group, and there were significant differences between Conn. D (*p* = 0.0355), Tb. N (*p* = 0.0017), Tb. Sp (*p* < 0.0001), and BMD (*p* = 0.0375) in the pre‐and post‐treatment results of the HPL group (Table 2). Furthermore, micro‐CT images showed sparse bone trabeculae in the superficial zone of OCAs after LPL and HPL treatments compared to the pre‐treatment OCAs (Figure 3).

**TABLE 2:** Comparison of the pre‐and post‐treatment OCAs Micro—Computed Tomography Evaluation

Group	Pre‐T BV/TV (%)	Post‐T BV/TV (%)	Pre‐T Conn. D (1/mm^3^)	Post‐T Conn. D (1/mm^3^)	Pre‐T Tb. N (1/mm)	Post‐T Tb. N (1/mm)	Pre‐T Tb. Sp (mm)	Post‐T Tb. Sp (mm)	Pre‐T BMD (mg HA/ccm)	Post‐T BMD (mg HA/ccm)
Saline	0.26 ± 0.06	0.25 ± 0.06	40.53 ± 2.70	40.97 ± 2.41	2.80 ± 0.20	2.80 ± 2.0	0.32 ± 0.03	0.32 ± 0.03	823.55 ± 15.76	830.29 ± 30.57
*p*‐value	0.8265		0.5232		0.5961		0.8093		0.6179	
Ultrasound	0.27 ± 0.02	0.28 ± 0.02	47.07 ± 6.38	47.41 ± 8.29	2.92 ± 0.16	2.89 ± 0.25	0.31 ± 0.02	0.31 ± 0.02	816.09 ± 9.36	813.96 ± 7.78
*p*‐value	0.1023		0.7485		0.6548		0.9599		0.1256	
VV	0.20 ± 0.02	0.20 ± 0.02	49.27 ± 10.44	47.60 ± 10.44	2.84 ± 0.24	2.85 ± 0.23	0.32 ± 0.03	0.32 ± 0.03	4851.32 ± 5.53	852.83 ± 4.40
*p*‐value	0.1396		0.3547		0.3945		0.4253		0.4412	
LPL	0.26 ± 0.03	0.24 ± 0.03	53.32 ± 3.50	52.76 ± 1.17	3.02 ± 0.14	2.96 ± 0.13	0.29 ± 0.02	0.31 ± 0.02	833.71 ± 14.10	829.43 ± 5.27
*p*‐value	0.0494*		0.7255		0.0060*		0.0145*		0.6010	
HPL	0.22 ± 0.30	0.21 ± 0.30	49.82 ± 6.28	47.24 ± 6.75	2.93 ± 0.18	2.82 ± 0.18	0.31 ± 0.02	0.32 ± 0.02	824.48 ± 7.73	841.05 ± 3.16
*p*‐value	0.1108		0.0355*		0.0017*		<0.0001*		0.0375*	
HSC	0.26 ± 0.06	0.26 ± 0.06	38.88 ± 2.53	39.40 ± 2.69	2.72 ± 0.13	2.68 ± 0.13	0.33 ± 0.02	0.34 ± 0.01	836.77 ± 13.52	834.51 ± 8.55
*p*‐value	0.2833		0.3552		0.1787		0.0853		0.7011	

*Note*: The values are expressed as mean ± SD unless otherwise indicated. The *p*‐value refers to the differences between the pre‐treatment and post‐treatment Micro‐Computed Tomography Evaluation.

Abbreviations: BMD, bone mineral density; BV/TV, bone volume per tissue volume; Conn. D, connection density of bone trabeculae; HPL, high‐pulse lavage; HSC, high‐speed centrifugation; LPL, low‐pulse lavage; OCAs, osteochondral allografts; Pre‐T, Pre‐treatment; Post‐T, Post‐treatment; Tb. N, trabecular number; Tb. Sp, trabecular space; VV, vortex vibration.

*P < 0.05 was considered statistically significant.

### Macroscopic Observations

**FIGURE 4:**
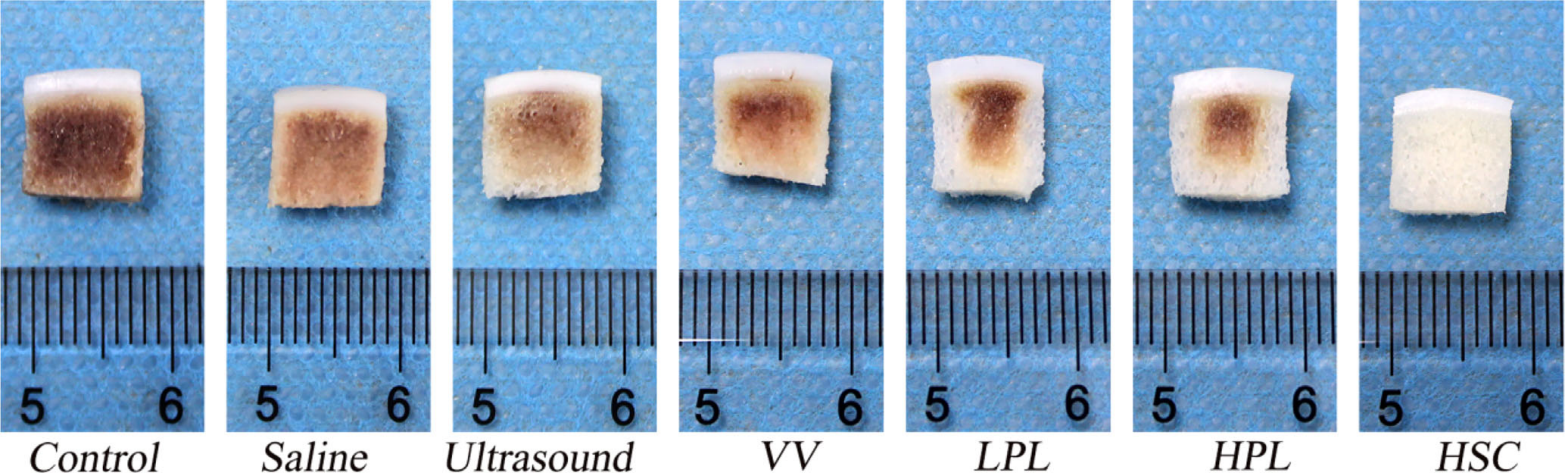
Gross photographs of OCAs after different method treatments. The trabeculae in the control group were all black. The peripheral trabeculae were yellow, and the central trabeculae were black in the saline, ultrasound, and VV groups. The peripheral trabeculae were white, and the central trabeculae were black in the LPL and HPL groups. The trabeculae in the HSC group were all white. HPL, high‐pulse lavage; HSC, high‐speed centrifugation; LPL, low‐pulse lavage; OCAs, osteochondral allografts; VV, vortex vibration.

Gross photographs of the OCAs showed that the trabeculae in the control group were all black, whereas in the saline, ultrasound, and VV groups, the peripheral trabeculae were yellow, and the central trabeculae were black. However, in the LPL and HPL groups, the peripheral trabeculae were white, and the central trabeculae were black. The most common characteristic was white trabeculae in the HSC group. Therefore, the HSC group exhibited the best macroscopic cleaning effect (Figure 4).

### Histological Findings

**FIGURE 5:**
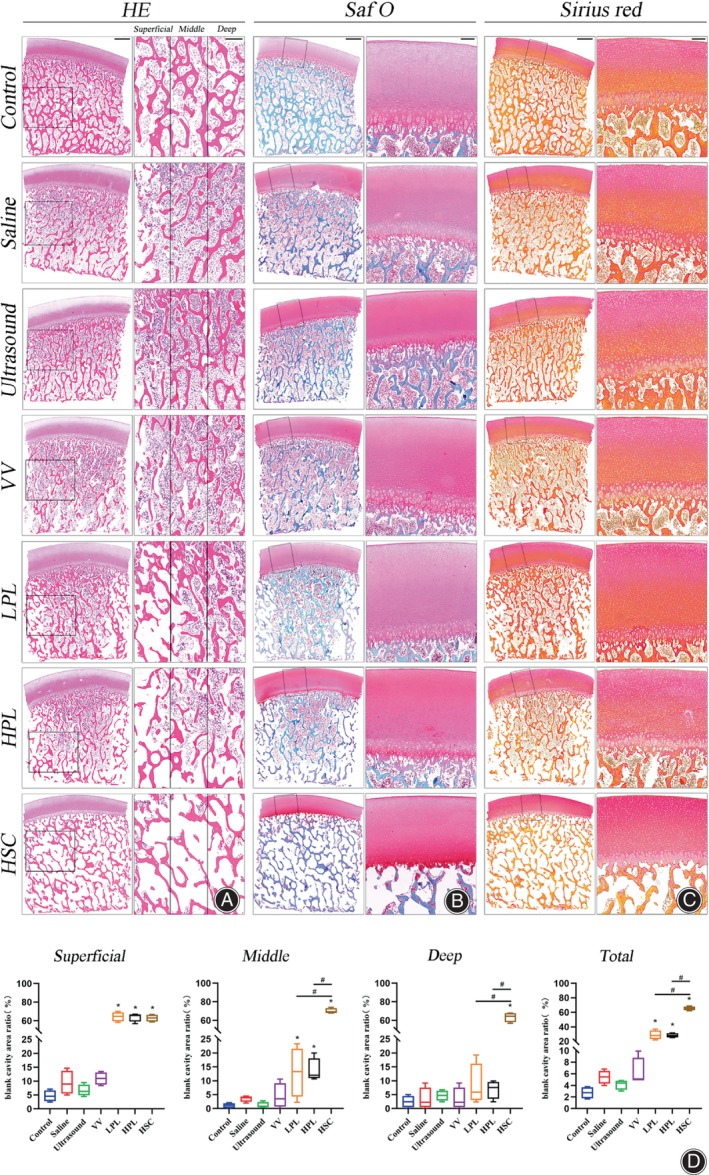
Histological assessment of OCAs. (A) Histological analysis by HE staining. Left‐side scale bar = 1000 μm and right‐side scale bar = 500 μm. (B) Histological analysis using Saf‐O staining. Left‐side scale bar = 1000 μm and right‐side scale bar = 200 μm. (C) Histological analysis using Sirius red staining. Left‐side scale bar = 1000 μm and right‐side scale bar = 200 μm. (D) Assessment of blank cavity area ratio of subchondral bone. Data are reported in box plots as mean, quartiles, and minimum and maximum values. Statistical analysis was performed using one‐way analysis of variance and the control and each treatment group were compared with Bonferroni's post‐hoc test (*p < 0.05), and the LPL, HPL, and HSC groups were compared with Bonferroni's post‐hoc test (#*p* < 0.05). HE, hematoxylin and eosin; HPL, high‐pulse lavage; HSC, high‐speed centrifugation; LPL, low‐pulse lavage; OCAs, osteochondral allografts; Saf‐O, Safranin‐O/fast green; VV, vortex vibration.

Histological results of the OCAs showed that Saf‐O staining confirmed the high preservation of GAG (Figure 5B), and Sirius red staining demonstrated type I/III COL preservation in cartilage tissues (Figure 5C). The blank cavity area ratio was analyzed in the different zones of the subchondral bone (Figure 5A). In the superficial zone, the blank cavity area ratio significantly increased in the LPL (64.40% ± 5.08%, *p* < 0.0001), HPL (63.84% ± 4.70%, *p* < 0.0001), and HSC (62.86% ± 3.77%, *p* < 0.0001) groups than in the control group (4.65% ± 1.98%). In the middle zone, the blank cavity area ratio significantly increased in the LPL (13.01% ± 8.88%, *p* = 0.0043), HPL (13.67% ± 4.27%, *p* = 0.0025), and HSC (70.13% ± 2.74%, *p* < 0.0001) groups compared to the control group (1.05% ± 0.85%). Additionally, the blank cavity area ratio was higher in the HSC group than in the LPL (*p* < 0.0001) and HPL (*p* < 0.0001) groups. In the deep zone, the blank cavity area ratio significantly increased in the HSC group (63.52% ± 5.09%, *p* < 0.0001) than in the control group (2.48% ± 2.08%). Further, the blank cavity area ratio was higher in the HSC group than in the LPL (8.37% ± 7.48%, *p* < 0.0001) and HPL groups (6.87% ± 3.26%, *p* < 0.0001). Overall, the blank cavity area ratio significantly increased in the LPL (28.60% ± 6.13%, *p* < 0.0001), HPL (28.13% ± 2.95%, *p* < 0.0001), and HSC (65.50% ± 2.73%, *p* < 0.0001) groups than in the control group (2.73% ± 0.99%). Furthermore, the blank cavity area ratio was higher in the HSC group than in the LPL (*p* < 0.0001) and HPL (*p* < 0.0001) groups (Figure 5D).

### DNA Quantification

**FIGURE 6:**
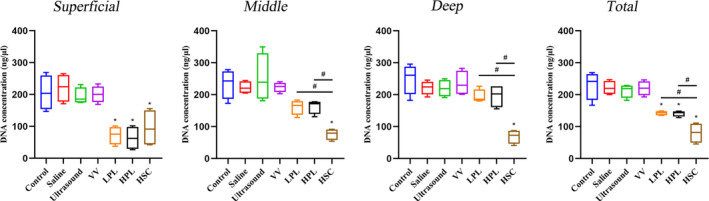
Evaluation of DNA concentration in different zones of OCAs. Data are reported in box plots with mean, quartiles, and minimum and maximum values. Statistical analysis was performed using one‐way analysis of variance and the control and each treatment group were compared with Bonferroni's post‐hoc test (*p < 0.05), and the LPL, HPL, and HSC groups were compared with Bonferroni's post‐hoc test (^#^ *p* < 0.05). HPL, high‐pulse lavage; HSC, high‐speed centrifugation; LPL, low‐pulse lavage; OCAs, osteochondral allografts; VV, vortex vibration.

The DNA concentration was also analyzed in the different zones of the subchondral bone (Figure 6). In the superficial zone, a remarkable reduction of DNA concentration in the LPL, HPL, and HSC groups was observed in the control group, with mean values of 72.58, 63.48, and 95.23 ng/μL, respectively, compared to 205.85 ng/μL in fresh OCAs, reaching reductions of 64.74% (*p* = 0.0010), 69.16% (*p* = 0.0005), and 53.74% (*p* = 0.0063). In the middle zone, a remarkable reduction of DNA concentration in the HSC groups was observed compared to the control group, with a mean of 76.48 ng/μL compared to 234.18 ng/μL in fresh OCAs, reaching 67.34% (*p* < 0.0001) reduction. Furthermore, the DNA concentration in the HSC group was lower than that in the LPL (*p* = 0.0009) and HPL (*p* = 0.0008) groups. In the deep zone, a remarkable reduction of DNA concentration in the HSC groups was observed compared to the control group, with a mean of 68.70 ng/μL compared to 249.90 ng/μL in fresh OCAs, reaching 72.52% (*p* < 0.0001) reduction. Additionally, the DNA concentration in the HSC group was lower than that in the LPL (*p* = 0.0003) and HPL (*p* = 0.0003) groups. Overall, a remarkable reduction of DNA concentration in the LPL, HPL, and HSC groups compared to the control group was observed, with a mean of 142.68, 140.99, and 80.14 ng/μL, respectively, compared to 229.98 ng/μL in fresh OCAs, reaching reductions of 37.96% (*p* = 0.0005), 38.69% (*p* = 0.0004), and 65.15% (*p* < 0.0001). Additionally, the DNA concentration in the HSC group was lower than that in the LPL (*p* = 0.0044) and HPL (*p* = 0.0053) groups. However, there were no significant differences in DNA concentrations among the saline, ultrasound, VV, or control groups (Figure 6).

### Chondrocyte Viability

**FIGURE 7:**
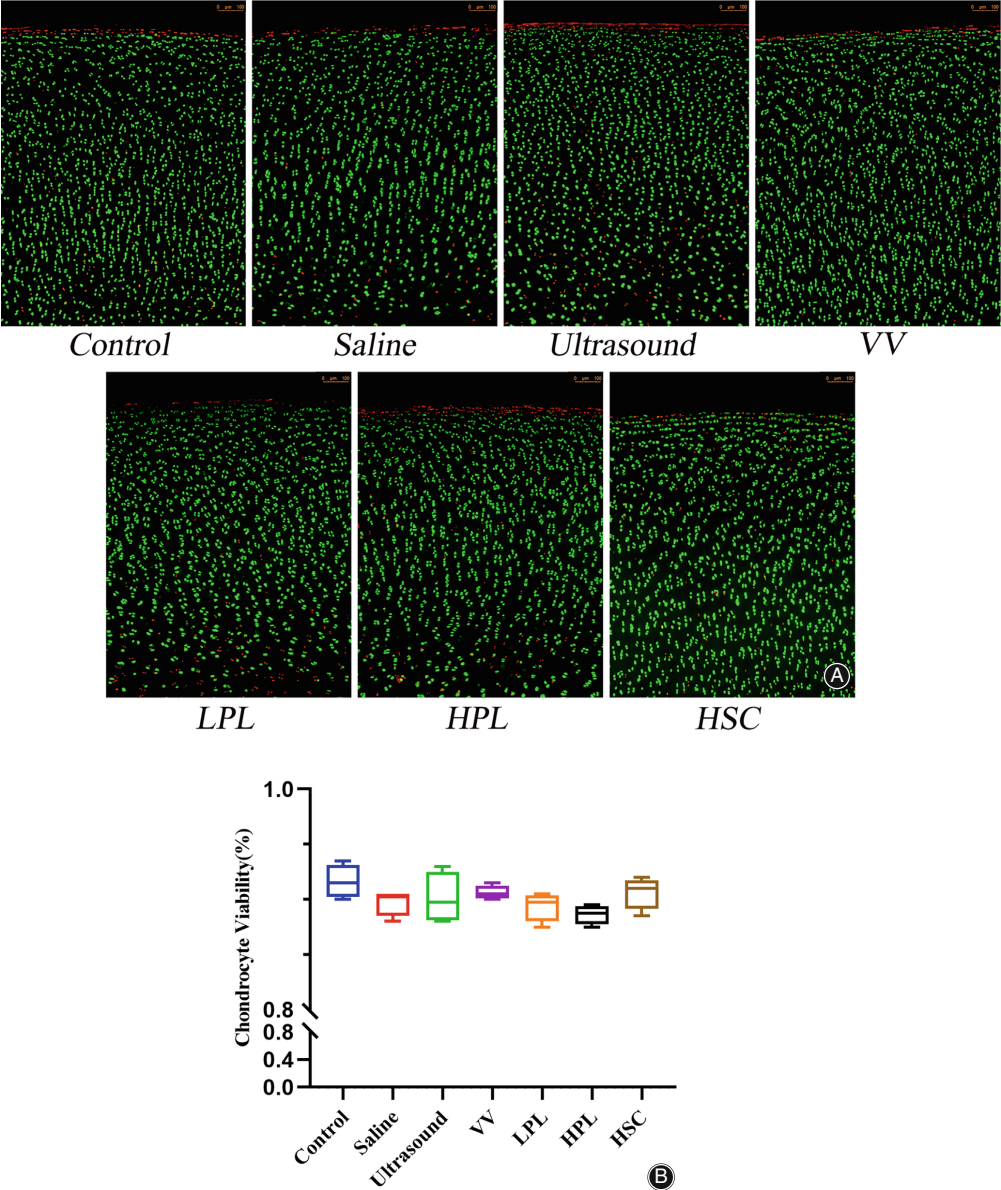
Assessment of chondrocyte viability. (A) The chondrocytes were stained; green for live cells, and red for dead cells. (B) Comparison of chondrocyte viability. The control and each treatment group were no statistical difference. Data are reported in box plots as mean, quartiles, and minimum and maximum values. Statistical analysis was performed using one‐way analysis of variance and the control and each treatment group were compared with Bonferroni's post‐hoc test (*p < 0.05). HPL, high‐pulse lavage; HSC, high‐speed centrifugation; LPL, low‐pulse lavage; OCAs, osteochondral allografts; VV, vortex vibration.

The staining results of live and dead cells showed that chondrocyte viability was higher than 80% in each group, although there was no statistical difference (Figure 7).

## Discussion

The results of this study indicated that HSC is the optimal pre‐transplant OCA treatment method that can reduce most immunogenic elements without destroying bone trabeculae and impairing chondrocyte activity and the extracellular matrix. Furthermore, our data showed the following: first, chondrocyte viability and extracellular matrix were not negatively affected after five methods of treating OCAs, but the PL technique may destroy bone trabecular structures; second, both PL and HSC can reduce the antigenic elements in the subchondral bone, with HSC being more effective in removing the superficial, medium, and deep zones antigenic elements of the subchondral bone. Additionally, after treatment of OCAs with HSC method, the bone‐marrow cavity blanking area could reach more than 60%, DAN content was reduced by 65%, and most of the bone marrow, lipids, and blood were removed.

### Importance of the Immunogenic Elements Removal in OCA

OCA transplantation presents an extremely special case among allogeneic transplant procedures because it does not require ABO blood type or human leukocyte antigen matching before transplantation.^19^ However, some OCA treatments before transplantation are necessary to reduce the entry of heterologous genes into the host. Moreover, as it is necessary to preserve chondrocyte activity and the integrity of the extracellular matrix of OCAs, chemical treatment is not an option, and physical treatment is preferred.

Generally, the success of OCA transplantation depends primarily on the integration of the graft‐host defect interface zone, where the integration and remodeling of the donor and host subchondral bone meet the biomechanical and biological requirements. Therefore, promoting the integration of the graft and host subchondral bone in the early post‐transplantation period is crucial. However, several studies have revealed that many immunogenic elements are present in the subchondral bone, such as blood, bone marrow, and lipids, which can cause an immune rejection reaction.^19^, ^20^, ^25^, ^36^, ^37^, ^38^ The immune rejection reaction has been confirmed in the primary subchondral bone after clinical OCA failure.^12^, ^13^, ^18^ Van Dijk proposed to minimize the bony portion of the graft, since creeping substitution is less effective over larger distances in thicker grafts.^39^ Additionally, overlong or excessive subchondral bone carries more immunogenic elements to produce a stronger immune response and does not allow for effective osseous integration.^17^, ^18^, ^20^, ^40^ A related study discovered that OCAs >9 mm thick were prone to bone cracks, which may be unfavorable for osseous integration.^6^ However, Babu et al.^20^ demonstrated that OCAs of 7 mm thickness resisted pullout significantly more than those of 4 mm thickness. Ackermann et al.^36^ discovered that OCAs with thinner thicknesses were more prone to subchondral cyst formation at the graft‐host junction, with bone thickness <5 mm increasing the risk of cystic degeneration nearly five‐fold. In this study, a subchondral bone thickness of 6 mm was used, consistent with the ideal subchondral bone thickness (3–6 mm).^6^, ^36^

### Limitations of PL Technique in OCA

PL technique is often used clinically to thoroughly wash the subchondral bone to reduce the blood and bone marrow content of the donor source.^4^, ^36^, ^41^ Meyer et al.^23^ proposed that combination saline and high‐pressure carbon dioxide lavage is more effective in removing bone marrow elements than saline PL alone. Sun et al.^24^ demonstrated that the effect of PL on fresh OCAs depends on irrigation intensity and time. However, Ambra et al.^19^ believed that PL could not effectively reduce the bone marrow content in OCAs; it could only wash the bone marrow in the superficial zone of the graft and might even concentrate the superficial bone marrow in the deep zone as the DNA content of OCAs did not show a significant decrease after PL. In this study, DNA quantification revealed that PL significantly reduced the bone marrow concentration in the superficial zone. Additionally, the gross photographs showed that the central area of the LPL and HPL groups was black and darker than that found in the saline, ultrasound, and VV groups (Figure 4). The authors attributed this to the fact that PL concentrates blood and bone marrow from the superficial zone of the OCAs in the deeper zone. Weight assessment showed a significant decrease in the weight of OCAs in the LPL and HPL groups; however, micro‐CT results revealed a significant difference between pre‐and post‐treatment with PL treatment (Table 2). Additionally, micro‐CT images showed sparse bone trabeculae in the superficial zone of OCAs after LPL and HPL treatments compared to the pre‐treatment OCAs (Figure 3). We attributed this to the fact that PL disrupts the superficial trabeculae of the subchondral bone, resulting in significant weight loss.

### Effectiveness and Potential of HSC Method in OCA

Centrifugation was often used to collect bone marrow from long bones.^26^, ^27^ This was the first study to use HSC to remove immunogenic elements from the subchondral bone. Gross photographs showed that the subchondral bone of the OCAs became white after 2 min, compared with the black color of the subchondral bone in the control group, and the black or yellow color of the subchondral bone in the other experimental group (Figure 4). Therefore, the authors concluded that HSC completely removed the blood element and effectively removed the lipid element. The lipid was present and appeared yellow in the bone trabeculae and in the degreased bones it became white.^25^ The histological and DNA quantification results showed a significant reduction in the bone marrow elements, and the HE results showed normal cell morphology; Saf‐O staining confirmed the high preservation of the GAG; and Sirius red staining demonstrated type I/III COL preservation in cartilage tissues in all groups (Figure 5). The residue (Figure S1.) in the 1.5‐mL centrifuge tube was subjected to frozen section and HE staining and was found to contain large amounts of lipids, blood, and various cells (Figure S2). The chondrocyte viability results showed that HSC treatment for 2 min did not damage the chondrocytes (Figure 7).

After zoning analysis of the subchondral bone was performed, and the histological and DNA quantification results showed that some bone marrow elements remained in the superficial zone of the subchondral bone after HSC, which the authors attributed to the absence of a dedicated HSC tube. Because the diameter of the OCAs was slightly smaller than that of the centrifuge tube, the subchondral bone clung to the wall of the tube, resulting in inadequate separation of the bone marrow in the superficial zone from the bone trabeculae. Moreover, the OCAs were centrifuged with a large amount of blood, lipids, and bone debris, which filled the space at the bottom of the tube, resulting in the bone marrow not being completely detached. To solve this problem, we designed a new centrifuge tube dedicated to centrifuging the OCAs to better remove the immunogenic elements of the subchondral bone and to apply larger OCAs. However, this study did not use the new tubes.

In studies relevant to the exploration of OCA failure and immune rejection, many methods have been proposed to suppress rejection, such as donor‐host antigen match, pre‐transplantation graft treatment methods, and post‐transplantation rejection suppression methods, all of which may directly or indirectly promote bone healing and integration of the graft and host. A clinical study discovered that sex mismatch transplantation may lead to OCA failure, particularly when male grafts are transplanted into female donors, with significantly lower 5‐year survival rates.^15^ Yang et al.^17^ used basic fibroblast growth factor and agarose gel‐coated OCAs implanted in homozygous rats to enhance immune privilege and showed that allografts and autologous transplantation produced the same healing effect. HSC could play an important role in decreasing immunogenicity and therefore has the potential to increase success.

### Limitation and Strengths

This study has some limitations. First, porcine knee joints were selected instead of human tissues because porcine joint size, cartilage thickness, and biomechanics are similar to those of humans and have a strong history of use for OCAs preservation experiments.^20^, ^25^, ^35^ Additionally, porcine osteochondral bone is also the most likely source of xenografts.^42^ However, differences in bone density and porosity of subchondral bone between humans and pigs may impact the results of this study. Second, OCAs in this study were only 8.5 mm in diameter. The thickness and size of the grafts may have affected the experimental results. Finally, we did not explore the effects of various centrifugation parameters such as speed and duration.

Nonetheless, this study has the following strengths. First, chondrocytes and the extracellular matrix were not destroyed after using these five physical methods for removing immunogenic elements. Second, these methods can be performed aseptically in a sterile environment. Thus, these methods are potentially the method of choice in decreasing immunogenicity for pre‐transplantation graft treatment.

## Conclusion

In summary, our study revealed that immunogenic elements were not significantly reduced after saline, ultrasound, or VV treatments of OCAs, whereas there was a significant reduction in immunogenic elements after LPL, HPL, and HSC treatments. Furthermore, HSC more effectively reduced the immunogenic elements of OCAs than LPL and HPL; blood, lipid, and bone marrow elements in the subchondral bone decreased significantly after HSC treatment. Additionally, micro‐CT, histological analyses, and chondrocyte viability results showed that HSC does not damage the subchondral bone and cartilage, whereas LPL and HPL may damage the subchondral bone. This study suggests that HSC has a potentially important role in decreasing immunogenicity and therefore may increase success in OCA transplantation.

## Author Contributions

Methodology, Conceptualization and Writing—Original Draft, Yongsheng Ma; Methodology, Qitai Lin and Wenming Yang; Validation, Yang Liu and Yugang Xing Formal Analysis, Zhiyuan Ren and Xueding Wang; Investigation, Raorao Zhou and Gaige Wu; Funding Acquisition, Pengcui Li and Xiaochun Wei; Supervision, Xiaoling Zhang; Writing—Review & Editing, Conceptualization and Funding Acquisition, Wangping Duan. All authors reviewed and approved the final submitted version.

## Conflict of Interest Statement

The authors declared no potential conflicts of interest with respect to the research, authorship, and/or publication of this article.

## Ethics Statement

All tissues were commercially sourced, no animals were specifically raised, bred, and sacrificed.

## Supporting information


**Figure S1.** OCA and residue after high‐speed centrifugation.
**Figure S2.** Antigen elements in centrifuge tubes by HE staining. The black arrows indicate lipids; the green arrow indicates blood; the yellow arrow indicates the cell.

## References

[1] Evuarherhe A Jr , Condron NB , Knapik DM , Haunschild ED , Gilat R , Huddleston HP , et al. Effect of mechanical mincing on minimally manipulated articular cartilage for surgical transplantation. Am J Sports Med. 2022;50(9):2515–2525. 35736385 10.1177/03635465221101004

[2] Jiang S , Guo W , Tian G , Luo X , Peng L , Liu S , et al. Clinical application status of articular cartilage regeneration techniques: tissue‐engineered cartilage brings new Hope. Stem Cells Int. 2020;2020:1‐16. 10.1155/2020/5690252 PMC7345961 32676118

[3] van Gastel N , Stegen S , Eelen G , Schoors S , Carlier A , Daniels VW , et al. Lipid availability determines fate of skeletal progenitor cells via SOX9. Nature. 2020;579(7797):111–117. 32103177 10.1038/s41586-020-2050-1 PMC7060079

[4] Hunt HE , Sadr K , Deyoung AJ , Gortz S , Bugbee WD . The role of immunologic response in fresh osteochondral allografting of the knee. Am J Sports Med. 2014;42(4):886–891. 24496509 10.1177/0363546513518733

[5] Du D , Hsu P , Zhu Z , Zhang C . Current surgical options and innovation for repairing articular cartilage defects in the femoral head. J Orthop Translat. 2020;21:122–128. 32309137 10.1016/j.jot.2019.06.002 PMC7152792

[6] Lai WC , Bohlen HL , Fackler NP , Wang D . Osteochondral allografts in knee surgery: narrative review of evidence to date. Orthop Res Rev. 2022;14:263–274. 35979427 10.2147/ORR.S253761 PMC9377395

[7] Crawford ZT , Schumaier AP , Glogovac G , Grawe BM . Return to sport and sports‐specific outcomes after osteochondral allograft transplantation in the knee: a systematic review of studies with at least 2 Years' mean follow‐up. Art Ther. 2019;35(6):1880–1889. 10.1016/j.arthro.2018.11.064 31053460

[8] Briggs DT , Sadr KN , Pulido PA , Bugbee WD . The use of osteochondral allograft transplantation for primary treatment of cartilage lesions in the knee. Cartilage. 2015;6(4):203–207. 26425257 10.1177/1947603515595072 PMC4568734

[9] Gilat R , Haunschild ED , Huddleston HP , Tauro TM , Patel S , Wolfson TS , et al. Osteochondral allograft transplant for focal cartilage defects of the femoral condyles: clinically significant outcomes, failures, and survival at a minimum 5‐year follow‐up. Am J Sports Med. 2021;49(2):467–475. 33428427 10.1177/0363546520980087

[10] Liu JN , Agarwalla A , Christian DR , Garcia GH , Redondo ML , Yanke AB , et al. Return to sport following high tibial osteotomy with concomitant osteochondral allograft transplantation. Am J Sports Med. 2020;48(8):1945–1952. 32459515 10.1177/0363546520920626

[11] Melugin HP , Ridley TJ , Bernard CD , Wischmeier D , Farr J , Stuart MJ , et al. Prospective outcomes of cryopreserved osteochondral allograft for patellofemoral cartilage defects at minimum 2‐year follow‐up. Cartilage. 2021;13(1_suppl):1014S–1021S. 32037873 10.1177/1947603520903420 PMC8808817

[12] Okeagu CN , Baker EA , Barreras NA , Vaupel ZM , Fortin PT , Baker KC . Review of mechanical, processing, and immunologic factors associated with outcomes of fresh osteochondral allograft transplantation of the talus. Foot Ankle Int. 2017;38(7):808–819. 28385038 10.1177/1071100717697649

[13] Wang T , Wang DX , Burge AJ , Pais M , Kushwaha B , Rodeo SA , et al. Clinical and MRI outcomes of fresh osteochondral allograft transplantation after failed cartilage repair surgery in the knee. J Bone Joint Surg Am. 2018;100(22):1949–1959. 30480599 10.2106/JBJS.17.01418

[14] Arzi B , DuRaine GD , Lee CA , Huey DJ , Borjesson DL , Murphy BG , et al. Cartilage immunoprivilege depends on donor source and lesion location. Acta Biomater. 2015;23:72–81. 26028293 10.1016/j.actbio.2015.05.025 PMC4522233

[15] Merkely G , Farina EM , Leite CBG , Ackermann J , Gortz S , Lattermann C , et al. Association of sex Mismatch between Donor and Recipient with Graft Survivorship at 5 years after osteochondral allograft transplantation. Am J Sports Med. 2022;50(3):681–688. 35044257 10.1177/03635465211068872

[16] Pomajzl RJ , Baker EA , Baker KC , Fleischer MM , Salisbury MR , Phillips DM , et al. Case series with histopathologic and radiographic analyses following failure of fresh osteochondral allografts of the talus. Foot Ankle Int. 2016;37(9):958–967. 27272267 10.1177/1071100716651963

[17] Yang F , Zhang Y , Liu B , Cao M , Yang J , Tian F , et al. Basic fibroblast growth factor and agarose gel promote the ability of immune privilege of allogeneic cartilage transplantation in rats. J Orthop Translat. 2020;22:73–80. 32440502 10.1016/j.jot.2019.07.001 PMC7231919

[18] Luk J , Stoker AM , Teixeiro E , Kuroki K , Schreiner AJ , Stannard JP , et al. Systematic review of osteochondral allograft transplant immunology: how we can further optimize outcomes. J Knee Surg. 2021;34(1):30–38. 33389738 10.1055/s-0040-1721670

[19] Ambra LF , de Girolamo L , Gomoll AH . Pulse lavage fails to significantly reduce bone marrow content in osteochondral allografts: a histological and DNA quantification study. Am J Sports Med. 2019;47(11):2723–2728. 31373832 10.1177/0363546519864716

[20] Babu JM , Hodax JD , Fadale PD , Owens BD . Osteochondral allograft transplantation: identifying the biomechanical impact of using shorter grafts and pulsatile lavage on graft stability. J Knee Surg. 2020;33(1):29–33. 30562831 10.1055/s-0038-1676501

[21] Haimi S , Wahlman M , Mannila M , Virtanen V , Hirn M . Pulse‐lavage washing is an effective method for defatting of morselized allograft bone in the operating theater. Acta Orthop. 2008;79(1):94–97. 18283579 10.1080/17453670710014824

[22] Ibrahim T , Qureshi A , McQuillan TA , Thomson J , Galea G , Power RA . Intra‐operative washing of morcellised bone allograft with pulse lavage: how effective is it in reducing blood and marrow content? Cell Tissue Bank. 2012;13(1):157–165. 21336569 10.1007/s10561-011-9241-9

[23] Meyer MA , McCarthy MA , Gitelis ME , Poland SG , Urita A , Chubinskaya S , et al. Effectiveness of lavage techniques in removing immunogenic elements from osteochondral allografts. Cartilage. 2017;8(4):369–373. 28934881 10.1177/1947603516681132 PMC5613898

[24] Sun Y , Jiang W , Cory E , Caffrey JP , Hsu FH , Chen AC , et al. Pulsed lavage cleansing of osteochondral grafts depends on lavage duration, flow intensity, and graft storage condition. PloS One. 2017;12(5):e0176934. 28464040 10.1371/journal.pone.0176934 PMC5413053

[25] Hua KC , Feng JT , Yang XG , Wang F , Zhang H , Yang L , et al. Assessment of the defatting efficacy of mechanical and chemical treatment for allograft cancellous bone and its effects on biomechanics properties of bone. Orthop Surg. 2020;12(2):617–630. 32189444 10.1111/os.12639 PMC7189055

[26] Amend SR , Valkenburg KC , Pienta KJ . Murine hind limb long bone dissection and bone marrow isolation. J Visual Exp: JoVE. 2016;110:53936. 10.3791/53936 PMC4941920 27168390

[27] Dobson KR , Reading L , Haberey M , Marine X , Scutt A . Centrifugal isolation of bone marrow from bone: an improved method for the recovery and quantitation of bone marrow osteoprogenitor cells from rat tibiae and femurae. Calcif Tissue Int. 1999;65(5):411–413. 10541770 10.1007/s002239900723

[28] Denbeigh JM , Hevesi M , Paggi CA , Resch ZT , Bagheri L , Mara K , et al. Modernizing storage conditions for fresh osteochondral allografts by optimizing viability at physiologic temperatures and conditions. Cartilage. 2021;13(1_suppl):280S–292S. 31777278 10.1177/1947603519888798 PMC8808875

[29] Harb A , von Horn A , Gocalek K , Schack LM , Clausen J , Krettek C , et al. Lactated ringer‐based storage solutions are equally well suited for the storage of fresh osteochondral allografts as cell culture medium‐based storage solutions. Injury. 2017;48(7):1302–1308. 28571706 10.1016/j.injury.2017.05.009

[30] Cook JL , Stoker AM , Stannard JP , Kuroki K , Cook CR , Pfeiffer FM , et al. A novel system improves preservation of osteochondral allografts. Clin Orthop Relat Res. 2014;472(11):3404–3414. 25030100 10.1007/s11999-014-3773-9 PMC4182376

[31] Qiao X , Zhang K , Li X , Lv Z , Wei W , Zhou R , et al. Gut microbiota and fecal metabolic signatures in rat models of disuse‐induced osteoporosis. Front Cell Infect Microbiol. 2022;12: 1018897. 10.3389/fcimb.2022.1018897 PMC9798431 36590590

[32] Zhong J , Mao X , Li H , Shen G , Cao X , He N , et al. Single‐cell RNA sequencing analysis reveals the relationship of bone marrow and osteopenia in STZ‐induced type 1 diabetic mice. J Adv Res. 2022;41:145–158. 36328744 10.1016/j.jare.2022.01.006 PMC9637485

[33] Stoker AM , Stannard JP , Kuroki K , Bozynski CC , Pfeiffer FM , Cook JL . Validation of the Missouri osteochondral allograft preservation system for the maintenance of osteochondral allograft quality during prolonged storage. Am J Sports Med. 2018;46(1):58–65. 28937783 10.1177/0363546517727516

[34] Liao S , Meng H , Zhao J , Lin W , Liu X , Tian Z , et al. Injectable adipose‐derived stem cells‐embedded alginate‐gelatin microspheres prepared by electrospray for cartilage tissue regeneration. J Orthop Translat. 2022;33:174–185. 35495963 10.1016/j.jot.2022.03.007 PMC9018217

[35] Wu K , Laouar L , Elliott JAW , Jomha NM . Vitrification of intact porcine femoral condyle allografts using an optimized approach. Cartilage. 2021;13(2_suppl):1688S–1699S. 33100019 10.1177/1947603520967077 PMC8721677

[36] Ackermann J , Merkely G , Shah N , Gomoll AH . Decreased graft thickness is associated with subchondral cyst formation after osteochondral allograft transplantation in the knee. Am J Sports Med. 2019;47(9):2123–2129. 31169995 10.1177/0363546519851098

[37] Baumann CA , Baumann JR , Bozynski CC , Stoker AM , Stannard JP , Cook JL . Comparison of techniques for preimplantation treatment of osteochondral allograft bone. J Knee Surg. 2019;32(1):97–104. 29514363 10.1055/s-0038-1636834

[38] Guder C , Gravius S , Burger C , Wirtz DC , Schildberg FA . Osteoimmunology: a current update of the interplay between bone and the immune system. Front Immunol. 2020;11:58. 32082321 10.3389/fimmu.2020.00058 PMC7004969

[39] van Dijk CN . Editorial commentary: bulk osteochondral Talar grafts compromise future arthrodesis or prosthesis. Art Ther. 2017;33(1):223–224. 10.1016/j.arthro.2016.11.002 28003071

[40] Ackermann J , Mestriner AB , Shah N , Gomoll AH . Effect of autogenous bone marrow aspirate treatment on magnetic resonance imaging integration of osteochondral allografts in the knee: a matched comparative imaging analysis. Art Ther. 2019;35(8):2436–2444. 10.1016/j.arthro.2019.03.033 31395183

[41] Cotter EJ , Christian DR , Frank RM , Abyar E , Wischmeier D , Yanke AB , et al. Survivorship of patellofemoral osteochondral allograft transplantation. Arthrosc Sports Med Rehabil. 2019;1(1):e25–e34. 32266337 10.1016/j.asmr.2019.06.003 PMC7120803

[42] Elder S , Chenault H , Gloth P , Webb K , Recinos R , Wright E , et al. Effects of antigen removal on a porcine osteochondral xenograft for articular cartilage repair. J Biomed Mater Res A. 2018;106(8):2251–2260. 29577591 10.1002/jbm.a.36411 PMC6779129

