# Supplementary material for: High‐Speed Centrifugation Efficiently Removes Immunogenic Elements in Osteochondral Allografts
Source: Orthop Surg. 2024 Jan 18;16(3):675–86. doi: 10.1111/os.13991 (PMC10925494; doi:10.1111/os.13991)
Supplement: Supplementary file 1 — Figure S1. OCA and residue after high‐speed centrifugation. Figure S2. Antigen elements in centrifuge tubes by HE staining. The black arrows indicate lipids; the green arrow indicates blood; the yellow arrow indicates the cell. [file OS-16-675-s001.docx]

**Preparation of Supplementary Data**


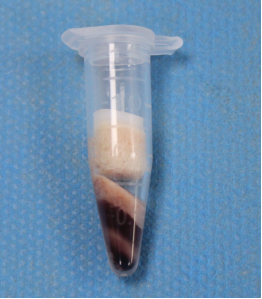


**Figure S1.** OCA and residue after high-speed centrifugation


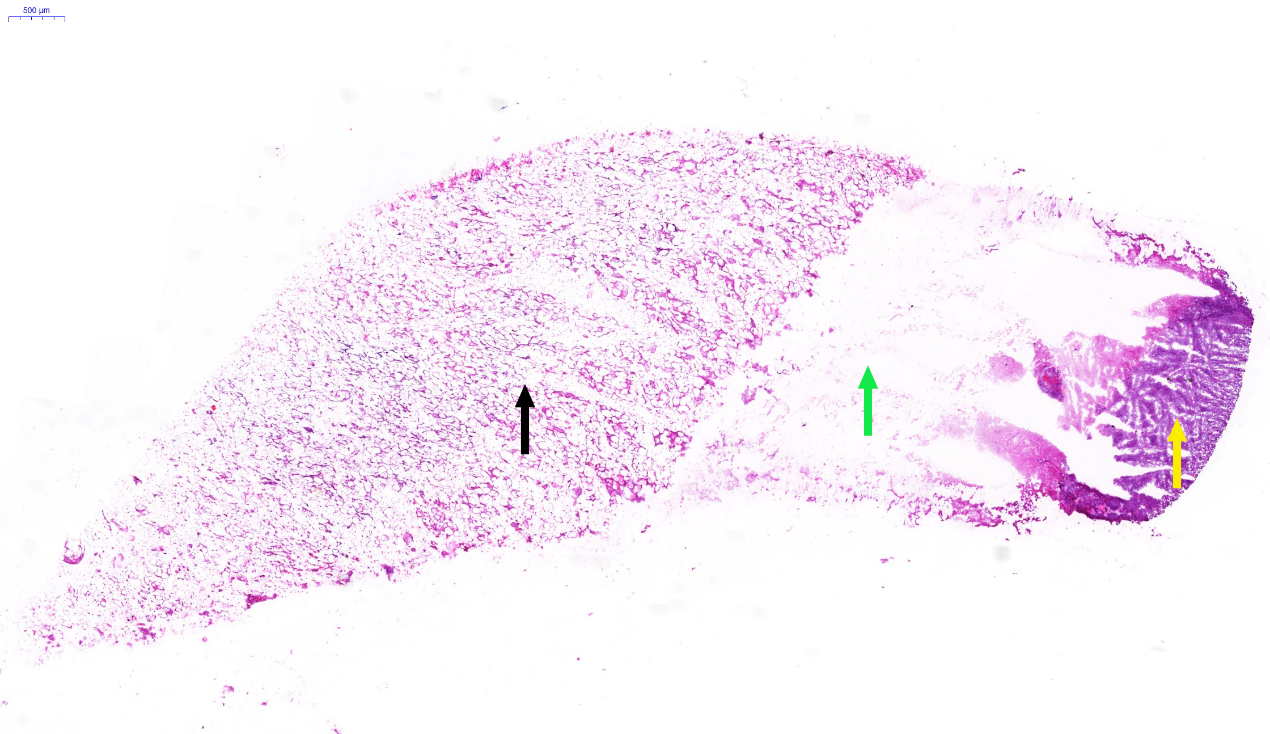


**Figure S2.** Antigen elements in centrifuge tubes by HE staining.

The black arrows indicate lipids; the green arrow indicates blood; the yellow arrow indicates the cell
